# Ultrasound cavitation: a reliable non-enzymatic method for adipose-derived mesenchymal stem cell (ADSC) isolation

**DOI:** 10.1186/s13287-023-03383-8

**Published:** 2023-06-06

**Authors:** Joo-Hoo Park, Yujin Choi, Jae-Min Shin, Hyun-Woo Yang, Seong-Ho Jeong, Il-Ho Park

**Affiliations:** 1 Upper Airway Chronic Inflammatory Diseases Laboratory, College of Medicine, Korea University, Seoul, Korea; 2 Department of Otorhinolaryngology-Head and Neck Surgery, Guro Hospital, Korea University College of Medicine, 80 Guro-Dong, Guro-Gu, Seoul, 152-703 Korea; 3 Medical Devices Usability Test Center, College of Medicine, Korea University, Seoul, Korea; 4 Department of Plastic Surgery, Guro Hospital, Korea University College of Medicine, 80 Guro-Dong, Guro-Gu, Seoul, 152-703 Korea

**Keywords:** Ultrasound cavitation, Adipose-derived stem cells, Non-enzymatic method

## Abstract

**Background:**

Adipose tissue is known to serve as an abundant and readily accessible source of adipose-derived stem cells (ADSCs) as an alternative to bone marrow. Collagenase is one of the most widely used methods for the isolation of ADSCs from adipose tissue, but it takes a long time, and there are also debates about safety. We propose an ultrasonic cavitation-treated method that can significantly reduce time and avoid the problem of using xenogeneic enzymes in ADSCs isolation.

**Methods:**

ADSCs were isolated from adipose tissue using the enzyme treatment method and the ultrasonic cavitation treatment method. Cell proliferation was measured using cell viability assay. The expression levels of the surface markers of ADSCs were estimated by real-time PCR. After, ADSCs were cultured in chondrogenic, osteogenic, or adipogenic differentiation medium; the differentiation potential of ADCSs was analyzed by Alcian blue, Alizarin Red S, Oil Red O, and real-time PCR.

**Results:**

The cells treated with collagenase and ultrasound had similar cell yields and proliferation after isolation. The difference in the expression of surface markers of ADSCs was not statistically significant. ADSCs showed differentiation potential into adipocytes, osteocytes, and chondrocytes, and there was no difference between the enzyme treatment method and the ultrasonic cavitation treatment method. The yield of the ADSC increased in time- and intensity dependently.

**Conclusions:**

Ultrasound certainly serves as a promising method in advancing ADSC isolation technology.

## Introduction

Adipose-derived stem cells (ADSCs) are mesenchymal stem cells (MSCs) isolated from white subcutaneous adipose tissue [1]. In recent years, adipose tissue has emerged as an alternative source of MSCs. Not only does adipose tissue give a higher MSC yield, but it also yields ADSCs through a less painful and minimally invasive extraction procedure compared with that used for bone marrow mesenchymal stem cells (BM-MSCs) [2]. ADSCs qualify as MSCs by meeting all three essential criteria: 1) cells adhere to plastic in culture; 2) cells express positive markers—CD105, CD73, and CD90—while not expressing negative markers—CD45, CD34, and CD31 or CD11b, CD79a, CD19, and HLA class II; and 3) cells can differentiate into mesoderm-derived cell types, osteoblasts, adipocytes, and chondroblasts [3]. The multipotency of ADSCs allows differentiated cells to be used in various tissue regeneration and reconstruction scenarios [4]. Currently, ADSCs are actively used in procedures for wound healing, immunomodulation in immune diseases such as graft-versus-host disease, and fat transfer in plastic surgery through adipose tissue extracted via liposuction [2].

Since its introduction in 2001, the procedure by Zuk et al., which uses collagenase II, has been one of the most widely used methods for ADSC isolation from adipose tissue [5]. The protocol involves lipoaspirate dissociation using collagenase II, followed by centrifugation to extract the stromal vascular fraction (SVF), which is a heterogeneous cell pellet consisting of “endothelial cells, erythrocytes, fibroblasts, lymphocytes, monocytes/macrophages, pericytes,” and ADSCs [6–8]. When SVF is cultured on a plastic plate, only ADSCs remain as the adherent cell population. There are concerns about the chemical side effects of using enzymes for cell processing, in addition to the time-consuming collagenase treatment and skepticism regarding the safety of treating cells with xenogeneic enzymes, even though there are animal-origin-free collagenase products available in the market for ADSC isolation [9].

To address this issue, multiple efforts to isolate ADSCs using non-enzymatic methods have been introduced, such as simply excluding enzymatic digestion or using mechanical force to isolate the cells [8]. While these methods demonstrate comparable performance to the common practice of using collagenase, none of them have been able to replace the existing practice, making the search for a minimally invasive and functionally equivalent, or even better, procedure for ADSC extraction, an ongoing task in the field of stem cell research [10]. Even among the non-enzymatic isolation techniques that have been suggested, ultrasound is a largely unexplored field despite its ubiquitous use in multiple research efforts and therapies in other fields. There have been a few efforts to utilize ultrasound in ADSC isolation, but mechanical isolation has always remained the main choice for non-enzymatic isolation techniques.

The use of ultrasound cavitation in treatment has been explored in various fields, such as drug delivery and tumor treatment, owing to its flexible application in cell permeabilization and the regulation of gene expression [11]. Ultrasound cavitation has also been widely used in plastic surgery for body contouring/fat reduction procedures [12]. Ultrasound cavitation separates cells through the continual expansion of bubbles as they encounter ultrasound waves, eventually causing the bubbles to burst, leading to cell separation [13]. Thus, ultrasound cavitation is a non-chemical means of separating cells without using collagenase [14]. This study shifts the focus toward the use of ultrasound cavitation in ADSC isolation. This technology is a promising alternative method for ADSC isolation, which can significantly decrease ADSC isolation time and void any concerns of using xenogeneic enzymes in ADSC isolation.

## Materials and methods

### Stem cell isolation

Lipoaspirates were obtained from healthy donors undergoing esthetic surgical procedures at the Department of Plastic Surgery of Korea University Guro Hospital. In accordance with the Declaration of Helsinki Principles, the study was conducted with written informed consent to collect human material samples from patients. Lipoaspirates were washed with phosphate-buffered saline (PBS) and disassembled using a gentleMACS™ Dissociator (Miltenyi Biotec, Bergisch Gladbach, Germany). For the enzymatic method, lipoaspirates were digested with 0.075% collagenase type I (Sigma-Aldrich, St. Louis, MO, USA) in Dulbecco’s modified Eagle’s medium (DMEM; Invitrogen, Carlsbad, CA, USA) with 10% fetal bovine serum (FBS; HyClone, Logan, UT, USA) and 1% penicillin/streptomycin (Invitrogen) for 1 h at 37 °C with constant shaking. The collagenase was neutralized using an equal volume of complete cell culture medium, which consisted of DMEM with 10% FBS and 1% penicillin/streptomycin. After centrifugation, the supernatant was removed from the SVF pellets and the SVF pellet was resuspended and the ADSCs were seeded in culture dishes in DMEM containing 10% FBS and 1% penicillin/streptomycin. For the non-enzymatic method, the disassembled lipoaspirates were treated with ultrasound cavitation using an Ultra stem cell (DMETEC Co., Ltd., Korea) at a power of 0 to 6 for 0 to 10 min to isolate the ADSCs. Subsequently, the digested lipoaspirates were filtered using a 100-µm filter (Merck-Millipore, Billerica, MA, USA) to collect the cell-containing media and centrifuged at 1000 g for 3 min to collect the SVF pellet. The SVF pellets were resuspended in DMEM, and the number of cells was counted using hemocytometer. Briefly, the cells were diluted in trypan blue solution and then added to the hemocytometer. The number of cells in each square of the hemocytometer was counted under a light microscope, and the total number of cells was calculated. The ADSCs were seeded in culture dishes in DMEM containing 10% FBS and 1% penicillin/streptomycin. The new fresh media were replaced every 2–3 days, and when the cells reach a sufficient number, we transfer them to a new flask and subculture for further use.

### Cell proliferation assay

Incucyte® cell count proliferation assay was performed to determine the cytotoxicity of butyric acid using the IncuCyte® cell count proliferation assay (Essen BioScience Inc., Ann Arbor, MI, USA). Briefly, the ADSCs were seeded in a 96-well plate at a density of 2 × 10^4^ cells per well. After seeding, the medium was replaced with 1% Incucyte® Nuclight Green BacMam 3.0 Reagent (Essen BioScience Inc.). The proliferation of the ADSCs cultured at 37 °C in an atmosphere containing 5% CO_2_ and 95% humidity was measured daily for 5 days. The viability of ADSCs was evaluated by measuring green fluorescence using the IncuCyte® software (Essen BioScience Inc.).

### CFU-F assay

CFU-F assay was performed to evaluate the colony-forming ability of adipose-derived stem cells (ADSCs). ADSCs were seeded at a density of 1.0 × 10^2^ cells/cm^2^ in a 6-well plate containing DMEM media supplemented with 10% fetal bovine serum (FBS) and 1% penicillin/streptomycin. The cells were incubated at 37 °C in a humidified atmosphere containing 5% CO_2_ for 14 days without changing the medium. After 14 days, the cells were fixed with 4% paraformaldehyde (PFA) for 10 min and then stained with 0.1% crystal violet for 30 min. The number of colonies containing more than 50 cells was counted using an inverted microscope.

### Multilineage differentiation of ADSCs

Briefly, 1 × 10^4^ cells/well of ADSCs were plated in 12-well tissue culture plates and cultured using osteogenesis, adipogenesis, and chondrogenesis differentiation kits (Gibco, Carlsbad, CA, USA). The medium was replaced every 3 days for 3 weeks. For osteogenic differentiation, the induced cells were analyzed via Alizarin Red S staining (ScienCell, Carlsbad, CA, USA) to assess mineralization. For adipogenic differentiation, the induced cells were stained with Oil Red O (Sigma, St. Louis, MO, USA). For chondrogenic differentiation, the induced cells were stained with Alcian blue (Sigma) to detect glycoproteins in the extracellular matrix. Images were obtained by an inverted microscope (Olympus CKX53; Olympus, Tokyo, Japan) and an upright microscope (Olympus BX43; Olympus, Tokyo, Japan).

### Real-time PCR

Total RNA was extracted using the TRIzol reagent (Invitrogen). cDNA was synthesized using the Maxime RT PreMix kit (Intron Biotechnology, Korea), according to the manufacturer’s protocol. An amplification reaction was performed with the following steps: an initial 2 min denaturation step at 94 °C; 40 cycles at 94 °C for 5 s, 60 °C for 10 s, and 72 °C for 20 s. All reactions were performed within a 20-μL volume. Real-time PCR was performed on the QuantStudio 3 system (Applied Biosystems, Foster City, CA, USA) with 100 ng cDNA template, 400 nM of each primer, and 10 μL Power SYBR Green PCR Master Mix (Applied Biosystems) in 20 μL. Relative gene expression was analyzed using the 2-ΔΔCT method. Each experiment was repeated at least three times, and GAPDH was used as the internal control. Forward and reverse primers used for PCR are shown in Table 1.

**Table 1:** Sequences of PCR primers

Gene name		Sequences (quantitative RT-PCR)
*CD90*	Forward	5ʹ-ATC GCT CTC CTG CTA ACA GTC-3ʹ
	Reverse	5ʹ-CTC GTA CTG GA TGG GTG AAC T-3ʹ
*CD29*	Forward	5ʹ-GTA ACC AAC CGT AGC AAA GGA-3ʹ
	Reverse	5ʹ-TCC CCT GAT CTT AAT CGC AAA AC-3ʹ
*CD105*	Forward	5ʹ-TGC ACT TGG CCT ACA ATT CCA-3ʹ
	Reverse	5ʹ-AGC TGC CCA CTC AAG GAT CT-3ʹ
*CD73*	Forward	5ʹ-CAG ATG TGG GAA GCT CCT GT-3ʹ
	Reverse	5ʹ-TGA CTG CTG GAA GTG GAG GT-3ʹ
*CD13*	Forward	5ʹ-GTG GAC AGC CTG AAG AAC TG-3ʹ
	Reverse	5ʹ-GGA GCT TTC AGA GAT GCC AG-3ʹ
*CD34*	Forward	5ʹ-TGG AAG GTT TGG ATC AGA GC-3ʹ
	Reverse	5ʹ-ACG GTC CTG CTT ATG GTG AT-3ʹ
*CD45*	Forward	5ʹ-CAC GTT AGC ACC CAC TTC AG-3ʹ
	Reverse	5ʹ-CCG TAG CTG CTT GTA GCC AT-3ʹ
*CD31*	Forward	5ʹ-GAA GAC AGG ATG GCT TCG AA-3ʹ
	Reverse	5ʹ-CCA GCC GTA GTG TCG TTG TA-3ʹ
*CD11b*	Forward	5ʹ-GCT GGA GGT GGA AAC TTG TC-3ʹ
	Reverse	5ʹ-TGG TCA CTC TTG GTG GTG TG-3ʹ
*CD19*	Forward	5ʹ-GAG AAC CCG GAG ACC TTT GA-3ʹ
	Reverse	5ʹ-TGC CGA GGT CTG TCT TCT TC-3ʹ
*FABP4*	Forward	5′-GCA TGG CCA AAC CTA ACA TGA-3′
	Reverse	5′-CCT GGC CCA GTA TGA AGG AAA-3′
*PPARγ*	Forward	5′-AGC CTC ATG AAG AGC CTT CCA-3′
	Reverse	5′-ACC CTT GCA TCC TTC ACA AGC-3′
*Sox9*	Forward	5ʹ-AGG TGC TCA AAG GCT ACG ACT-3ʹ
	Reverse	3ʹ-AGA TGT GCG TCT GCT CCG TG-5ʹ
*Col2A*	Forward	5′-ACT TGC GTC TAC CCC AAT CC–3′
	Reverse	5′-ACA GTC TTG CCC CAC TTA CC-3′
*Runx2*	Forward	5′-CCA CGA CAA CCG CAC CAT-3′
	Reverse	5′-CAC TCC GGC CCA CAA ATC TC-3′
*Osteocalcin*	Forward	5′-CAC TCC TCG CCC TAT TGG C-3′
	Reverse	5′-CCC TCC TGC TTG GAC ACA AAG-3′
*GAPDH*	Forward	5′-GTG GAT ATT GTT GCC ATC AAT GAC C-3′
	Reverse	5′-GCC CCA GCC TTC TTC ATG GTG GT-3′

### Statistical analysis

The data are presented as the means ± standard deviation of experiments, repeated at least three times, and analyzed by GraphPad Prism 5 (Graph Pad Software, San Diego, CA, USA) with paired *t*-tests, unpaired *t*-tests or one-way analysis of variance, and Tukey’s test. Each experiment was conducted triplicate. *p* values < 0.05 were considered statistically significant difference.

## Results

### Comparison of cell yield and proliferation from isolation with collagenase and ultrasound methods

**Fig. 1:**
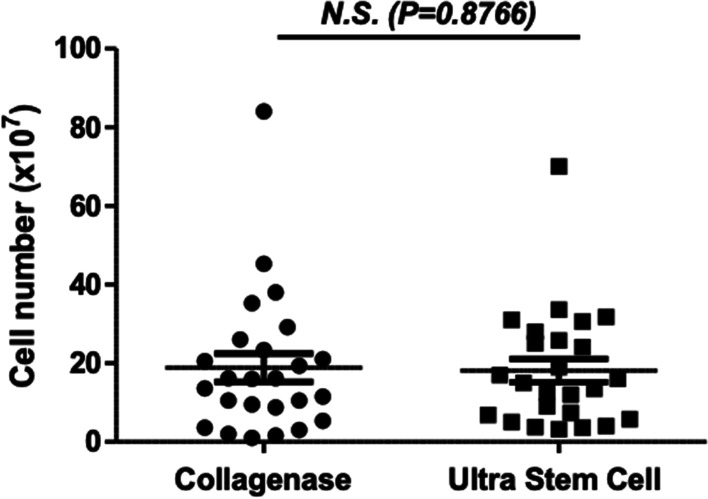
Number of isolated MSCs (per the amount initially in the adipose tissue sample) after samples are treated with collagenase and ultrasound methods. Data are expressed as mean ± SEM. *p < 0.05 versus control

The collagenase and ultrasound methods yielded a similar number of isolated cells. The median cell yield for both methods was approximately 2 × 10^8^ cells/ml, and the upper and lower quartiles were also similar for both methods (Fig. 1).

**Fig. 2:**
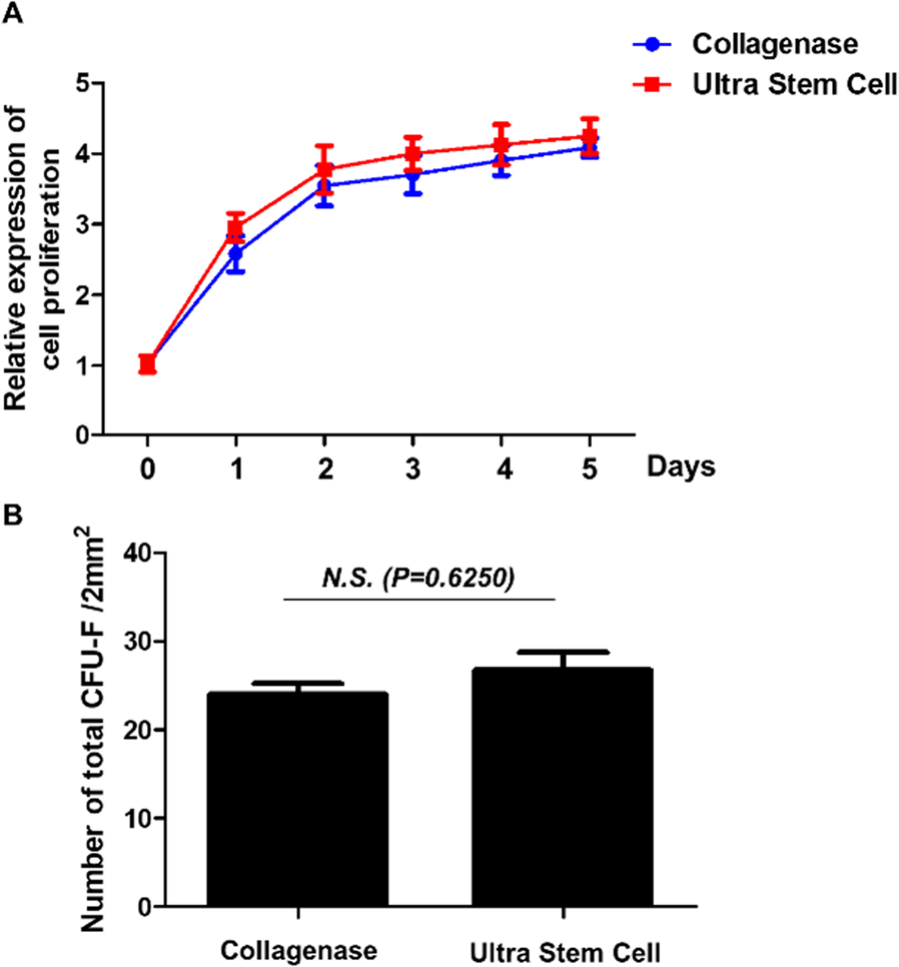
**A** Cell proliferation was measured using the Incucyte® cell count proliferation assay. The fluorescence values are presented relative to the control as shown on the Y-axis. **B** The proliferation of cells treated with collagenase and ultrasonic cavitation was evaluated using the CFU-F assay. Data are expressed as mean ± SEM

Cell proliferation, measured relative to day 0, was slightly higher for cells obtained using the ultrasound method. However, the growth trends between the collagenase and ultrasound methods were very similar, and the difference in cell proliferation and CFU-F was minimal (Fig. 2A and B).

### Characterization of adipose-derived stem cells isolated using ultrasound

**Fig. 3:**
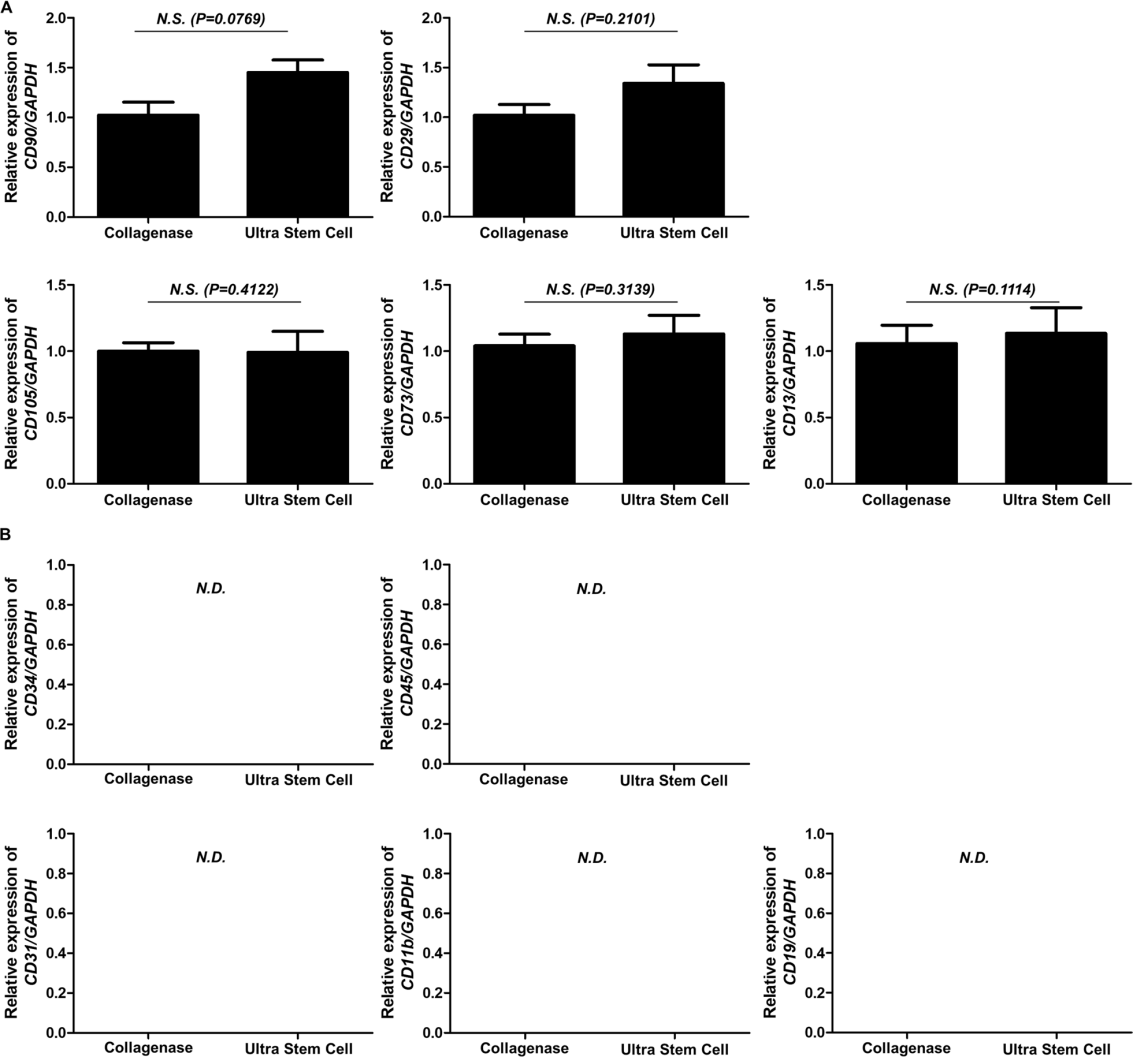
Real-time PCR results of positive and negative markers on cells treated with collagenase and ultrasound. Presented here is the relative expression of positive markers **A** CD90, CD29, CD105, CD73, and CD13 and negative markers **B** CD34, CD45, CD31, CD11b, and CD19 compared with that of GAPDH. Data are expressed as mean ± SEM

The expression levels of positive markers were higher in ultrasound-treated samples. While with the collagenase-treated cells, the relative expression levels of CD90 (Thy-1), CD29 (Integrin beta-1), CD105 (Endoglin), CD73 (Ecto-5'-nucleotidase), and CD13 (Aminopeptidase N) were approximately 1, the ultrasound-treated cells had relative expression levels of approximately 1.3. However, this difference was not statistically significant (Fig. 3A). Additionally, CD34 (hematopoietic progenitor cell antigen CD34), CD45 (leukocyte common antigen), CD31 (platelet endothelial cell adhesion molecule-1), CD11b (integrin alpha-M), and CD19 (B-lymphocyte antigen CD19) markers were not identified in either treatment sample, which was expected because CD34, CD45, CD31, CD11b, and CD19 are negative markers (Fig. 3B).

### Multilineage differentiation of adipose-derived stem cells

**Fig. 4:**
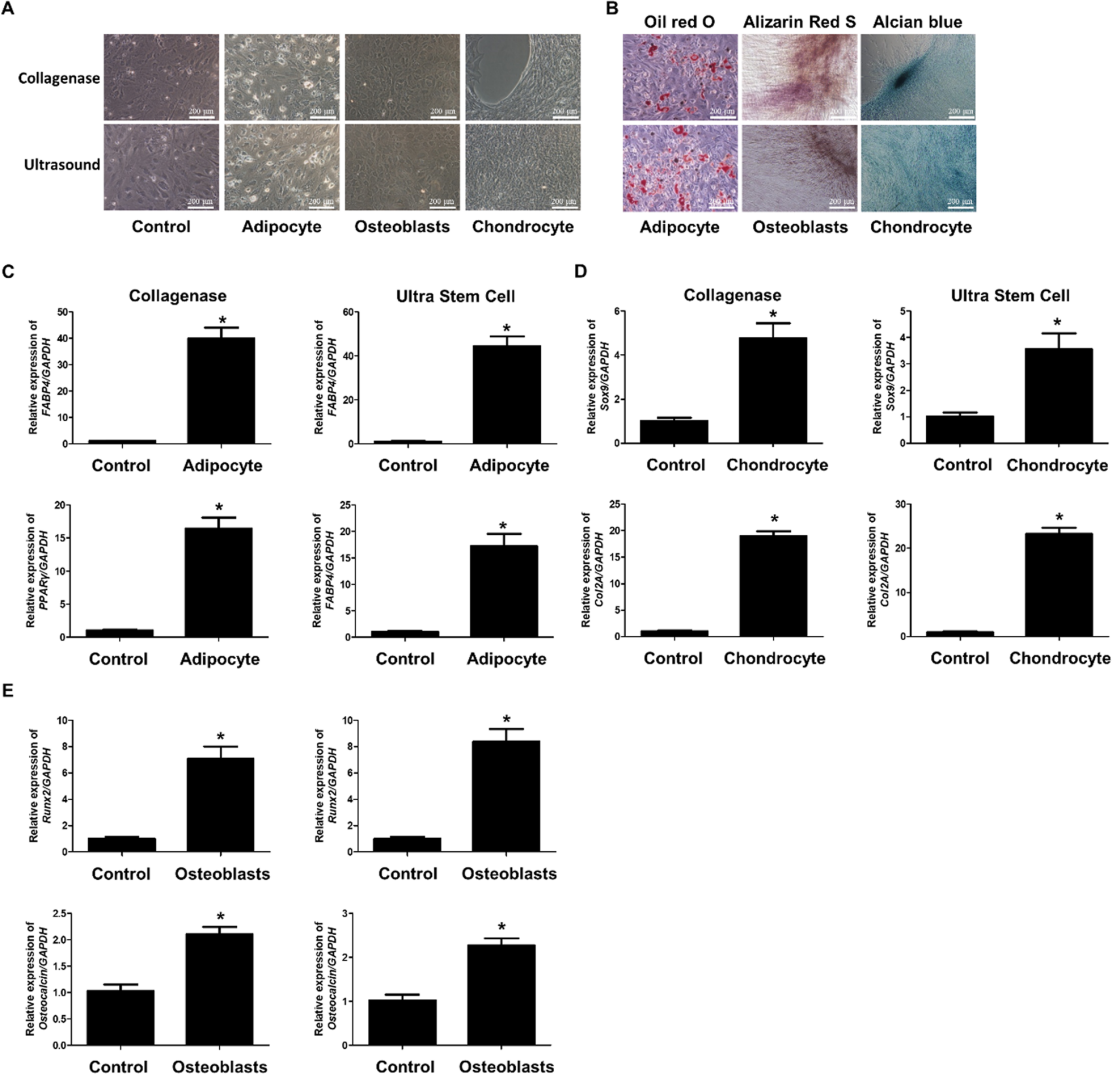
Marker levels of MSCs and adipocytes differentiated from collagenase-treated cells. Cell microscopy (left) and staining (right) images of control (MSCs) and differentiated cells for collagenase- and ultrasound-treated cells (**A**). Adipocytes were stained with Oil Red O, osteoblasts were stained with Alizarin Red S, and chondrocytes were stained with Alcian blue (**B**). The expression of two adipocyte differentiation markers (FABP4 and PPARγ) in MSCs and adipocytes differentiated from collagenase- or ultrasound-treated cells was measured using real-time PCR (**C**). The expression of two chondrocyte differentiation markers (Sox9 and Col2A) in MSCs and chondrocytes differentiated from collagenase- or ultrasound-treated cells was measured using real-time PCR (**D**). The expression of two osteocyte differentiation markers (Runx2 and Osteocalcin) in MSCs and osteocytes differentiated from ultrasound- or collagenases-treated cells were measured using real-time PCR (**E**). Data are expressed as mean ± SEM of three independent samples. *p < 0.05 versus control

These were then observed under a microscope. Both collagenase- and ultrasound-treated cells successfully differentiated into each cell type (Fig. 4A and B). The mRNA expression of adipocyte differentiation markers (FABP4 and PPARγ), chondrocyte differentiation markers (Sox9 and Col2A), and osteocyte differentiation markers (Runx2 and osteocalcin) was increased in each type of differentiated cell (Fig. 4C, D, and E).

### Further settings for ultrasound

**Fig. 5:**
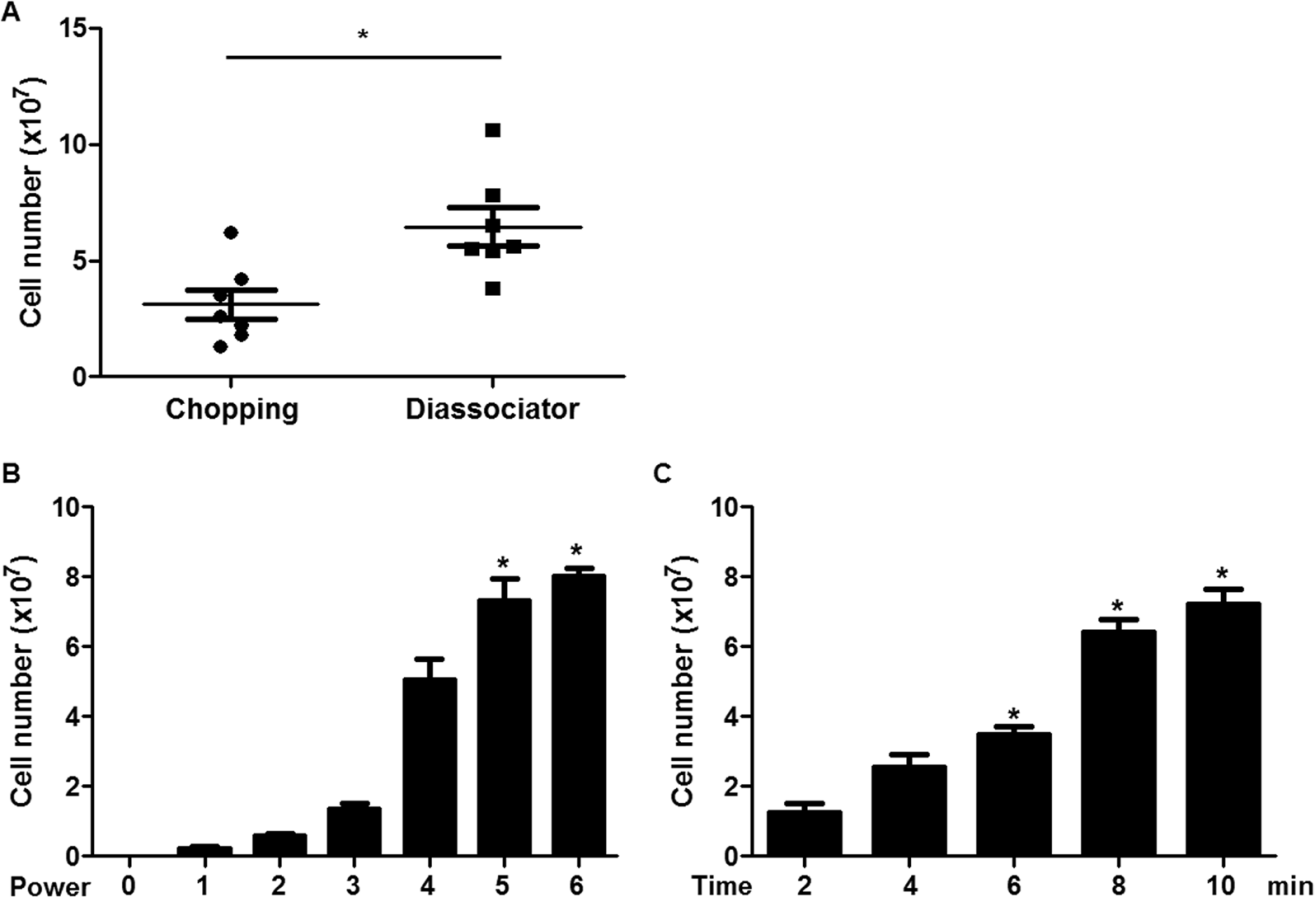
MSC cell count for samples that were either chopped or placed in a dissociator. Cells were treated with ultrasound afterward for MSC isolation (**A**). Number of isolated MSCs based on different power (**B**) and time settings (**C**) on the ultrasound machine. Data are expressed as mean ± SEM of seven independent samples. *p < 0.05 versus control

We confirmed the difference in yield when the adipose tissue is chopped before ultrasound treatment, which is the current method, versus that when the tissue is ground in the dissociator. When the tissue was homogenized using a dissociator, a standard deviation of 3.114 ± 0.6356 was obtained, while cutting the tissue finely resulted in a higher yield with a standard deviation of 6.457 ± 0.8280, representing approximately twice the yield compared to the dissociator method (Fig. 5A). Different ultrasound intensities and treatment times were evaluated. The ADSCs yield was greatest when ultrasound was applied at the highest intensity (power 6) and for 10 min (Fig. 5B and C).

## Discussion

Efficient isolation methods for adipose-derived stem cells, which are widely used in clinical and cosmetic applications, have been studied [15]. While enzymatic methods have been commonly used, non-enzymatic methods, particularly ultrasound-based techniques, have been proposed but not yet widely adopted [16, 17]. According to the results of our study, both collagenase and ultrasound methods were equally effective in isolating adipose-derived stem cells, showing similar cell yield and growth trends. However, the ultrasound-treated samples showed higher expression levels of positive markers, although this difference was not statistically significant. Both collagenase and ultrasound-treated cells successfully differentiated into each cell type and exhibited increased expression of differentiation markers. Our study also suggests that finely chopping the adipose tissue before ultrasound treatment leads to a higher cell yield compared to homogenization using a tissue dissociator. Based on cell yield and proliferation, the collagenase and ultrasound methods appear to show similar levels of performance in isolating ADSCs from adipose tissue. Sufficient yield and proliferation of cells indicated that ADSCs were successfully extracted and adhered to the culture plate, satisfying the first criteria for cells to qualify as functional ADSCs.

While protein expression of the positive stem cell markers CD90, CD29, CD105, CD73, and CD13, and the absence of negative stem cell markers CD34, CD45, CD31, CD11b, and CD19 should ideally be confirmed using flow cytometry, this study was limited to verifying marker expression at the mRNA level only [18]. Despite this limitation, the results show to indirectly confirm the characteristics of ADSCs by assessing the presence or absence of marker expression at the mRNA level. As expected, the expression of positive stem cell markers CD90, CD29, CD105, CD73, and CD13, and the absence of the expression of negative stem cell markers CD34, CD45, CD31, CD11b, and CD19, was observed. This confirmed that only ADSCs remained in the sample after the isolation process. Interestingly, it should be noted that ultrasound-treated cells had higher expression levels of both positive stem cell markers, CD90, CD29, CD105, CD73, and CD13, than collagenase-treated cells. In addition to these differences in marker expression, the number of CFU-Fs that may be characteristic of ADSCs, although not significant, tended to increase when ultrasonic cavitation was used [19]. Therefore, it suggests the possibility that ADSCs that were isolated using ultrasound cavitation are more potent than ADSCs isolated using collagenase. However, since the difference in relative expression did not exceed 0.5 and there are other possible factors that could affect the levels of stem cell markers and the number of CFU-Fs, further consideration is required before making this claim.

MSCs have characteristics to differentiate into multiple cell lineages such as chondrocytes, osteocytes, adipocytes, and neuron-like cells in vitro [20]. Comparing differentiation markers, all six markers for adipocytes, chondrocytes, and osteocytes demonstrated similar levels of expression in ultrasound- and collagenase-isolated ADSCs. The considerable expression levels of all six differentiation markers in ultrasound- and collagenase-isolated cells suggest successful differentiation of the isolated ADSCs into all three cell types. Thus, both methods are able to yield functional ADSCs with intact differentiation capabilities.

In addition, it appears that the cell yield only increases as the intensity and exposure time to ultrasound increase within the capability of the ultrasound machine. However, further testing at higher intensities and longer exposure times beyond the scope of the machine could be beneficial in determining the optimal setting at which ultrasound cavitation will best yield ADSCs. The preservation of cell functionality must also be confirmed for the respective intensities and exposure times.

ADSCs are typically isolated from adipose tissue using enzymatic digestion with collagenase. However, there has been some debate in the scientific community regarding the use of collagenase, particularly due to concerns about the potential negative effects of the enzyme on the cells [21]. When using adipose-derived stem cells isolated by enzymes for clinical applications, there is a concern that heterologous substances may remain in the cells in small amounts, which could potentially have negative effects [22]. Collagenase is known to be cytotoxic and can degrade extracellular matrix components and disrupt cell–cell interactions, which could potentially affect the viability, proliferation, and differentiation potential of ADSCs [23]. Despite these concerns, during the past decades some animal-origin-free collagenase has been introduced to the market and many studies have successfully reported the isolation of different tissue-specific MSCs with them [22]. Nevertheless, non-enzymatic techniques such as ultrasound cavitation have been continuously investigated as an alternative method that can further decrease the isolation time of ADSCs compared to enzymatic methods and alleviate concerns about potential side effects.

## Conclusion

Given these concerns regarding collagenase use, ultrasound offers a significant advantage as it is not attributed with any concerns regarding cell behavior change or xenogenic chemical use. The fact that ultrasound can produce comparable, if not improved, performance to collagenase in isolating ADSCs offers a very compelling alternative. Not only does the ultrasound technique prevent any concern for maintaining cGMP in the isolation process, but it also performs at a level that is comparable with the level of the ADSC isolation technique that is currently the most widely used. Moreover, as it has other advantages such as decreasing ADSC isolation time and introducing a xeno-free method for ADSC isolation, ultrasound certainly serves as a promising method in advancing ADSC isolation technology.

## Data Availability

All data generated or analyzed during this study are included in this published article.

## Abbreviation

ADSCs: Adipose-derived stem cells
MSCs: Mesenchymal stem cells
BM-MSCs: Bone marrow mesenchymal stem cells
SVF: Stromal vascular fraction
PBS: Phosphate-buffered saline
FBS: Fetal bovine serum
cGMP: Common good manufacturing practices
HCT/P: Human cells, tissues, or cellular or tissue-based products
